# Supplementary material for: Kinetic Aspects of Benzene Degradation over TiO2-N and Composite Fe/Bi2WO6/TiO2-N Photocatalysts under Irradiation with Visible Light
Source: Int J Mol Sci. 2023 Mar 16;24(6):5693. doi: 10.3390/ijms24065693 (PMC10051460; doi:10.3390/ijms24065693)
Supplement: Supplementary file 1 [file ijms-24-05693-s001.zip › ijms-2207137-supplementary.pdf]

Article

Kinetic Aspects of Benzene Degradation over TiO_2-N and Composite Fe/Bi_2WO_6/TiO_2-N Photocatalysts under Irradiation with Visible Light

Mikhail Lyulyukin [1,2], Nikita Kovalevskiy [1], Andrey Bukhtiyarov [1], Denis Kozlov [1] and Dmitry Selishchev [1,*]

1 Boreskov Institute of Catalysis, Novosibirsk 630090, Russia
2 Ecology and nature management department, Aircraft engineering faculty, Novosibirsk State Technical University, Novosibirsk 630073, Russia
* Correspondence: selishev@catalysis.ru; Tel.: +7-383-326-9429

Supplementary Materials

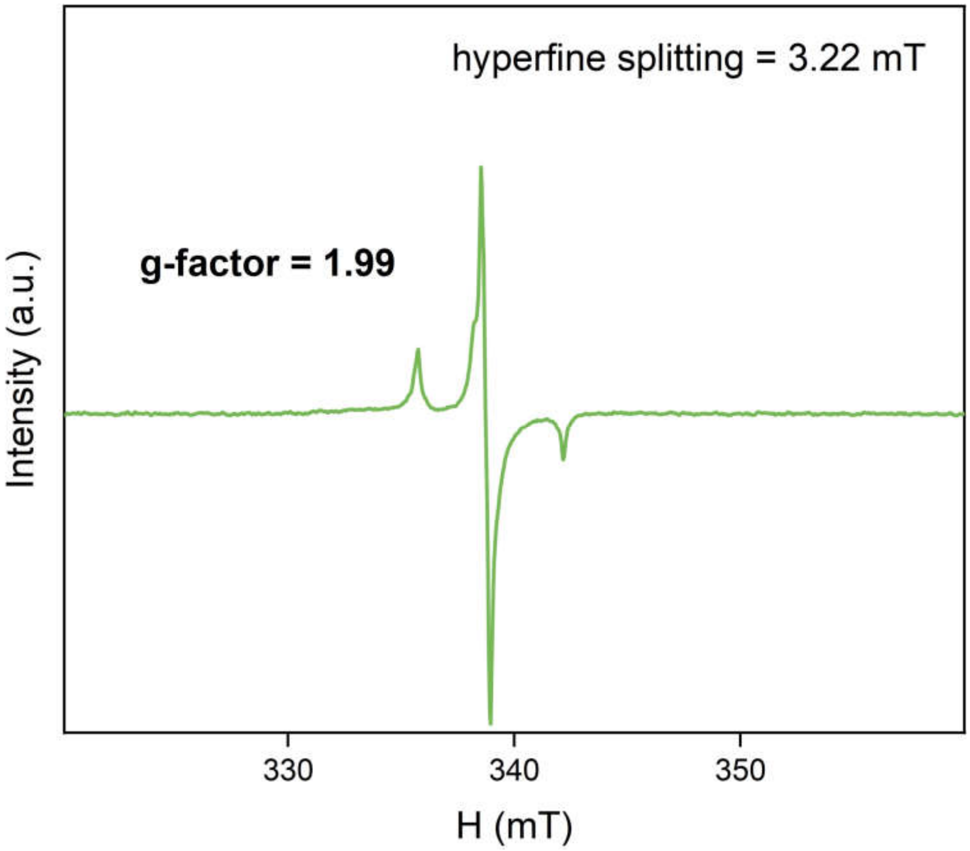

Figure S1. EPR signal of paramagnetic N centers in the synthesized TiO_2-N photocatalyst.

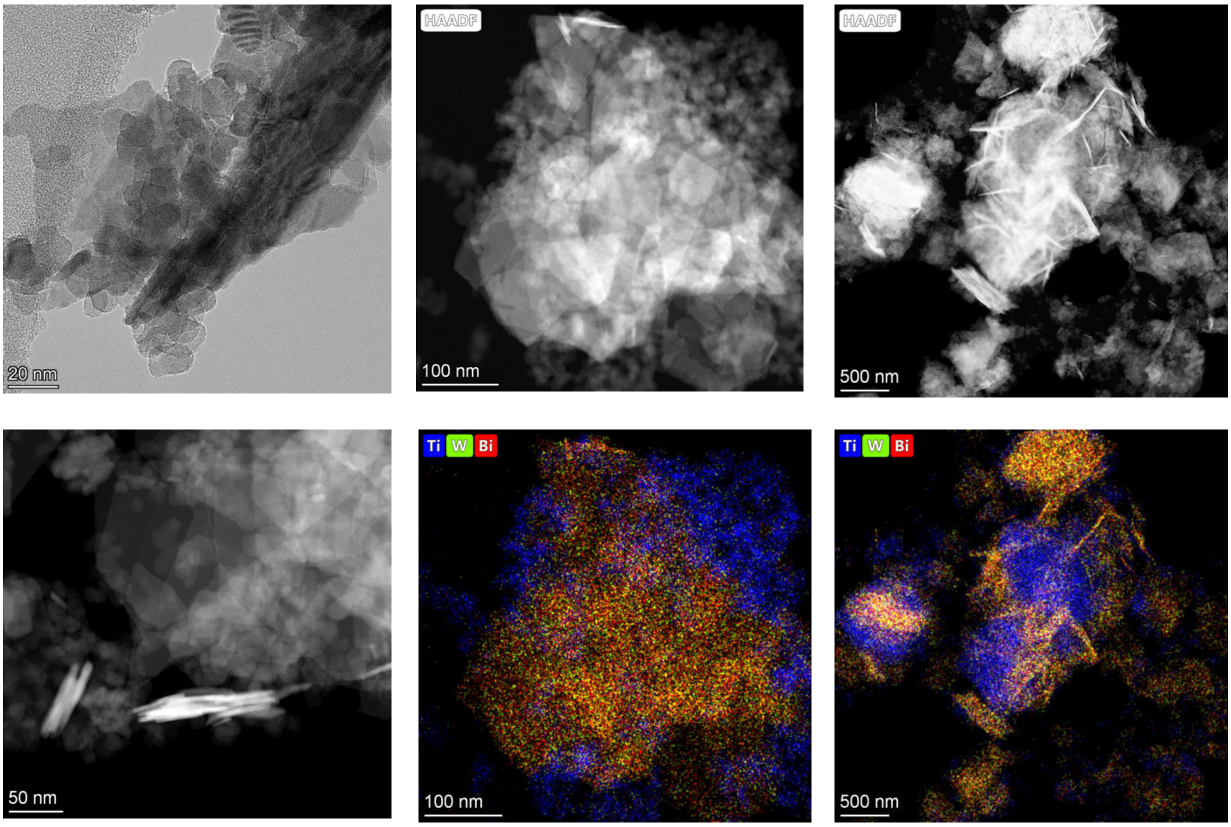

Figure S2. TEM micrographs of Bi_2WO_6/TiO_2-N obtained using HAADF imaging and EDX mapping techniques.

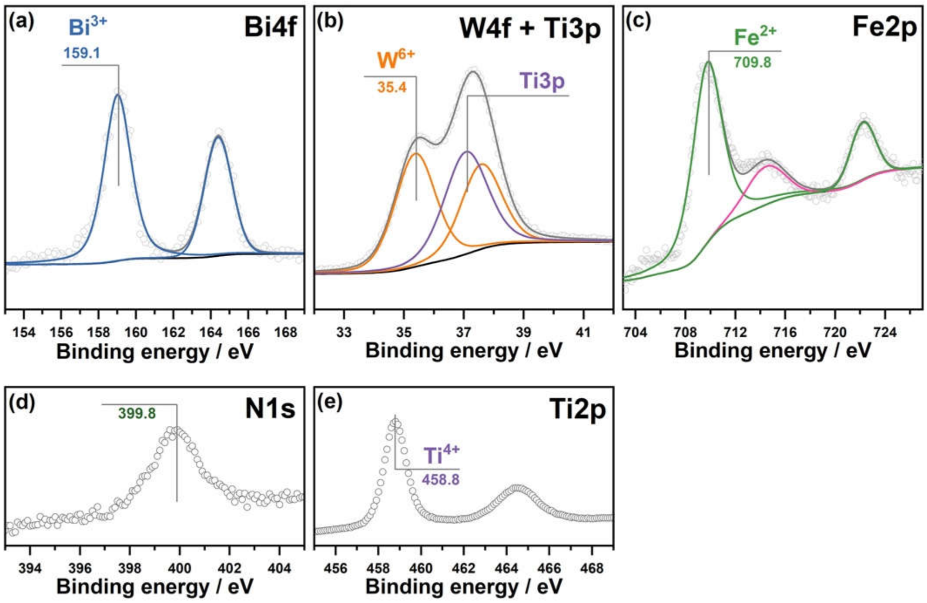

Figure S3. Photoelectron Bi4f (**a**), W4f+Ti2p (**b**), Fe2p (**c**), N1s (**d**), and Ti2p (**e**) spectral regions for the synthesized 0.3Fe/Bi_2WO_6/TiO_2-N sample.

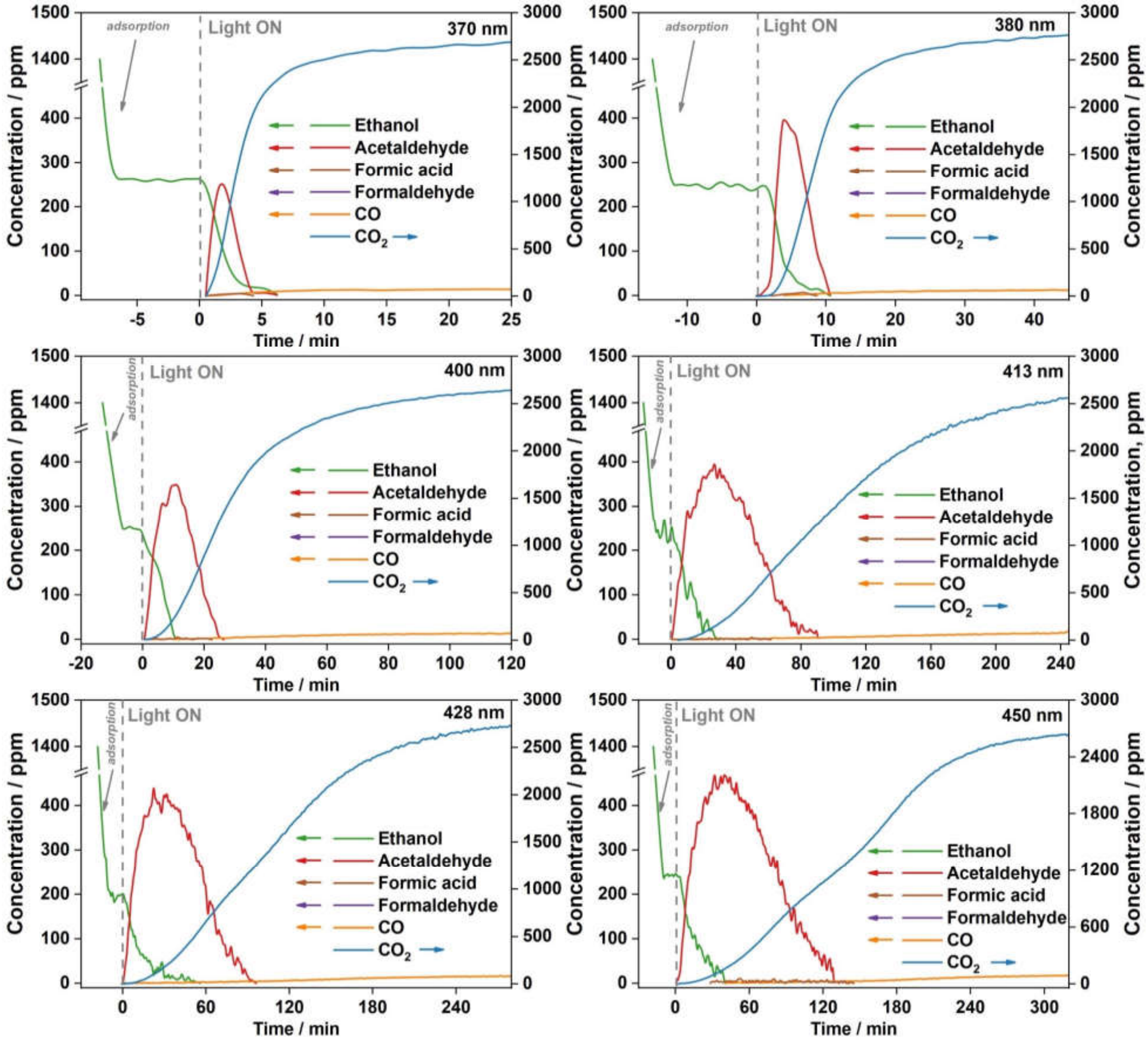

Figure S4. Kinetic plots of the reaction components during PCO of ethanol over TiO_2-N under irradiation with a maximum at: 370, 380, 400, 413, 428, and 450 nm.

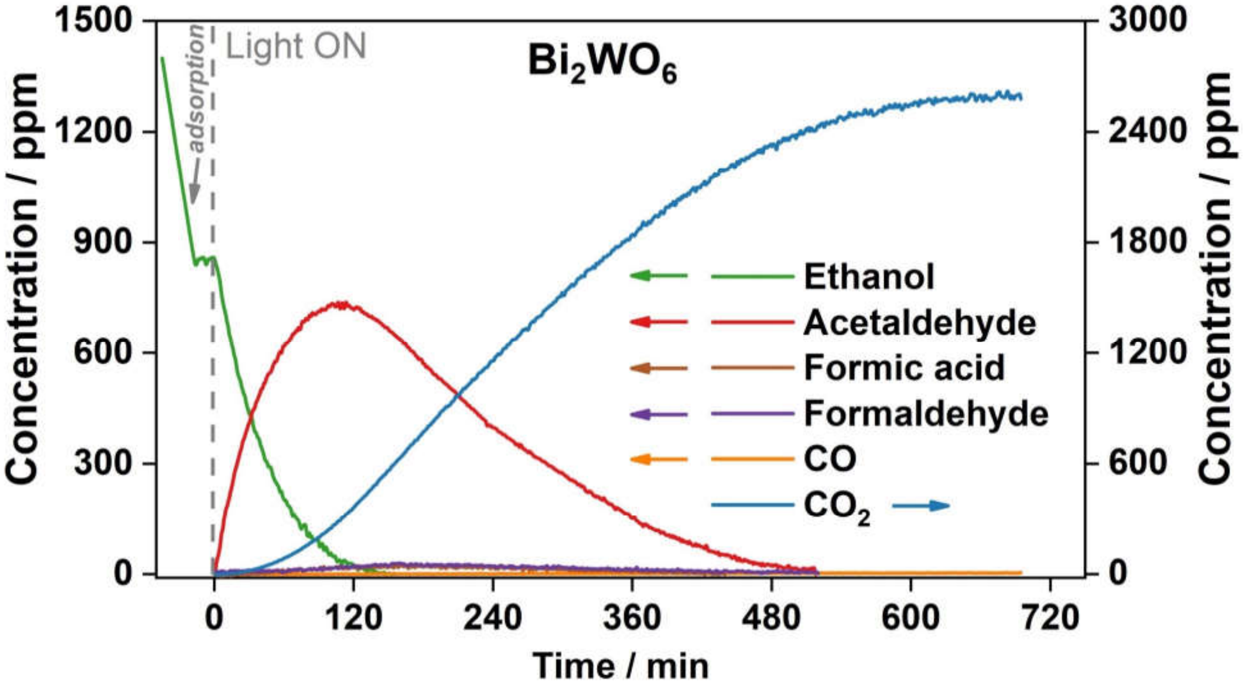

Figure S5. Kinetic plots of the reaction components during PCO of ethanol over Bi_2WO_6 under blue light.

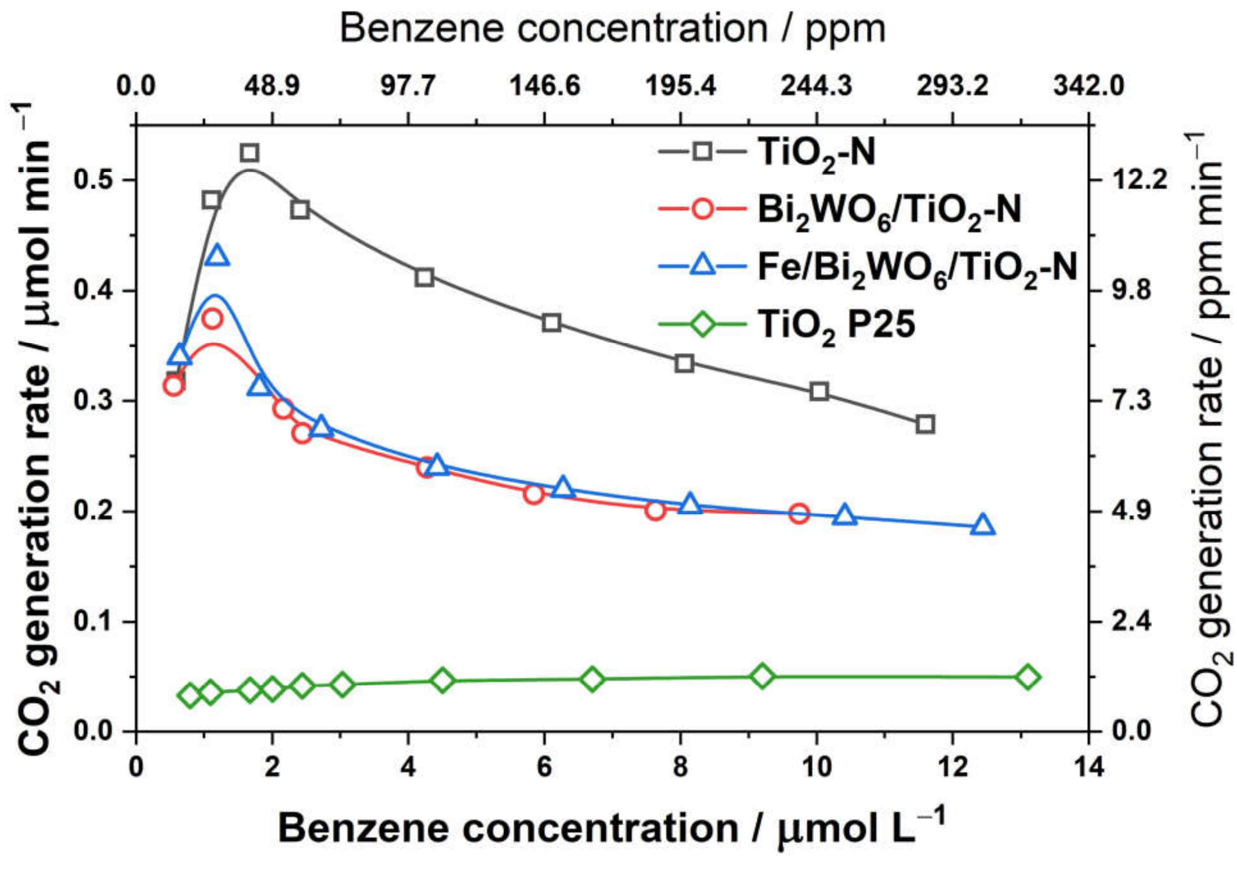

Figure S6. Effect of inlet benzene concentration on the rate of CO_2 formation during PCO of benzene over TiO_2-N, Bi_2WO_6/TiO_2-N, Fe/Bi_2WO_6/TiO_2-N, and Evonik TiO_2 P25 under blue light.

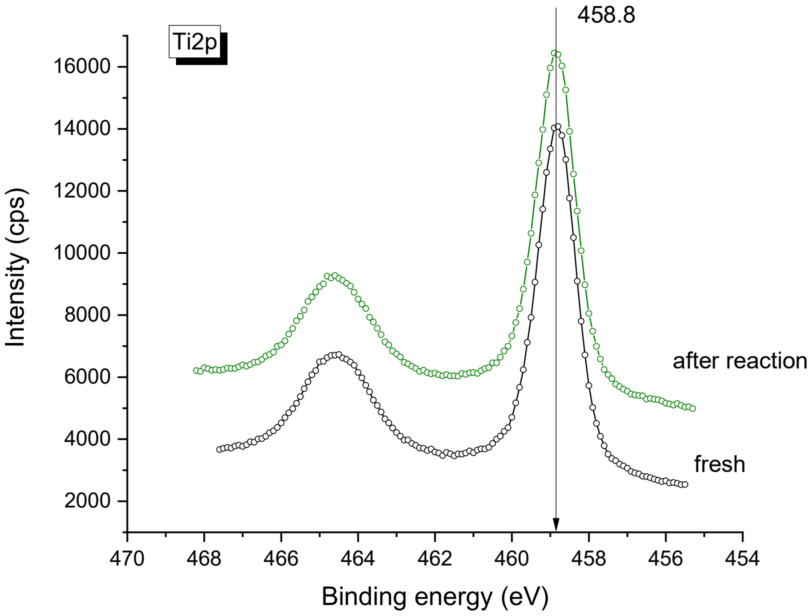

Figure S7. Ti2p core-level spectra for the TiO_2-N catalyst before and after the stability test.

Table S1. Atomic concentrations (%) of the elements on the surface of TiO_2-N before and after the stability test.

	**C**	**N**	**Ti**	**O**
Before reaction	40.5	0.5	16.6	42.5
After reaction	40.6	0.4	17.4	41.6
